# Dynamic changes in kynurenine pathway metabolites in multiple sclerosis: A systematic review

**DOI:** 10.3389/fimmu.2022.1013784

**Published:** 2022-11-02

**Authors:** Mobina Fathi, Kimia Vakili, Shirin Yaghoobpoor, Arian Tavasol, Kimia Jazi, Ashraf Mohamadkhani, Andis Klegeris, Alyssa McElhinney, Zahedeh Mafi, Mohammadreza Hajiesmaeili, Fatemeh Sayehmiri

**Affiliations:** ^1^ Student Research Committee, Faculty of Medicine, Shahid Beheshti University of Medical Sciences, Tehran, Iran; ^2^ Faculty of Medicine, Medical University of Qom, Qom, Iran; ^3^ Liver and Pancreatobiliary Diseases Research Center, Digestive Disease Research Institute, Shariati Hospital, Tehran University of Medical Sciences, Tehran, Iran; ^4^ Department of Biology, Faculty of Science, University of British Columbia, Kelowna, BC, Canada; ^5^ Faculty of Medicine, Shahid Beheshti University of Medical Sciences, Tehran, Iran; ^6^ Critical Care Quality Improvement Research Center, Loghman Hakim Hospital, Shahid Beheshti University of Medical Sciences, Tehran, Iran

**Keywords:** multiple sclerosis, kynurenine pathway, kynurenine, tryptophan, picolinic acid, hydroxyindoleacetic acid, kynurenic acid, quinolinic acid

## Abstract

**Background:**

Multiple sclerosis (MS) is a debilitating neurodegenerative disorder characterized by axonal damage, demyelination, and perivascular inflammatory lesions in the white matter of the central nervous system (CNS). Kynurenine pathway (KP), which is the major route of tryptophan (TRP) metabolism, generates a variety of neurotoxic as well as neuroprotective compounds, affecting MS pathology and the severity of impairments. Alterations in KP have been described not only in MS, but also in various psychiatric and neurodegenerative diseases. The purpose of this systematic review is to investigate the previously reported dysregulation of KP and differences in its metabolites and enzymes in patients with MS compared to healthy control subjects.

**Method:**

Electronic databases of PubMed, Scopus, Cochrane Database of Systematic Reviews, and Web of Science were searched to identify studies measuring concentrations of KP metabolites and enzymes in MS patients and control subjects. The following metabolites and enzymes implicated in the KP were investigated: TRP, kynurenine (KYN), kynurenic acid (KYNA), quinolinic acid (QUIN), picolinic acid (PIC), hydroxyindoleacetic acid (HIAA), indoleamine 2,3-dioxygenase (IDO), kynurenine aminotransferase (KAT), and their related ratios.

**Result:**

Ten studies were included in our systematic review. Our review demonstrates that IDO expression is reduced in the peripheral blood mononuclear cells (PBMCs) of MS patients compared to healthy controls. Also, increased levels of QUIN and QUIN/KYNA in the serum and cerebrospinal fluid (CSF) of MS patients is observed. Differences in levels of other metabolites and enzymes of KP are also reported in some of the reviewed studies, however there are discrepancies among the included reports.

**Conclusion:**

The results of this investigation suggest a possible connection between alterations in the levels of KP metabolite or enzymes and MS. QUIN levels in CSF were higher in MS patients than in healthy controls, suggesting that QUIN may be involved in the pathogenesis of MS. The data indicate that differences in the serum/blood or CSF levels of certain KP metabolites and enzymes could potentially be used to differentiate between MS patients and control subjects.

## Introduction

Multiple sclerosis (MS) is one of the most prevalent neurological disorders worldwide, with an annual incidence rate of approximately 2 per 100,000 (1). MS is a disabling neurodegenerative, autoimmune, inflammatory, and demyelinating disease of the central nervous system (CNS) (2), predominantly affecting young adults during their most productive years which is from 20 to 50 (3, 4).

MS is characterized by axonal damage, demyelination, and perivascular inflammatory lesions in the CNS white matter. T lymphocytes autoreactive against CNS antigens may initiate MS pathogenesis (5). Numerous proinflammatory factors and cytokines have been found to be altered in the blood, brain tissues, and cerebrospinal fluid (CSF) of MS patients (6). The kynurenine pathway (KP) in MS is induced by proinflammatory cytokine cascades resulting in altered levels of KP metabolites (7, 8).

The KP is critical for providing cellular energy to the immune system under physiological conditions, by generating nicotinamide adenine dinucleotide (NAD^+^). However, the metabolites of KP with neuroactive function, collectively referred to as “kynurenines” play an important role in chronic neuroinflammation. Under inflammatory conditions, these metabolites are typically considered neurotoxic and gliotoxic due to their adverse effects on glutamatergic neurotransmission and direct toxicity towards neurons and glial cells (9, 10). In addition, tryptophan (TRP) and some intermediate metabolites in the KP exhibit immunomodulatory properties. It is also well established that the indoleamine 2,3-dioxygenase (IDO) enzyme significantly contributes to immune regulation by depleting TRP and producing kynurenine (KYN) (11, 12). A link between the aryl hydrocarbon receptor and IDO is identified in the expansion of Th17 and regulatory T cells, which plays a significant role in various autoimmune disorders and cancer (13, 14). Kynurenic acid (KYNA), which is a metabolite produced through the KP, acts as a neuroprotective agent, while quinolinic acid (QUIN) is an established neurotoxic agent (15–20). Overall, alterations in the KP and in TRP metabolism are critical in MS pathogenesis, since abnormalities in TRP metabolism have been shown to impair regulation of T cell activity (21).

In the KP, TRP is the first substrate converted to KYN by two enzymes named IDO and tryptophan-2,3-dioxygenase (TDO**)**. KYN is subsequently catalyzed by kynurenine aminotransferase (KAT) and kynurenine-3-monooxygenase (KMO) to produce two different metabolites, 3-hydroxykynurenine (3-HK) and KYNA, respectively. 3-HK is altered to 3-hydroxyanthranillic acid (3-HANA**)** by an enzyme called kynureninase, and the next metabolite produced from 3-HANA is QUIN. At last, NAD^+^ is the ultimate metabolite of TRP produced through the KP (22).

While activation of some KP enzymes have short-term benefits, such as decreased T cell proliferation and immunosuppression, their chronic activation results in the production of neurotoxic metabolites and impairs the innate repair mechanism of remyelination (23). In MS patients, proinflammatory cytokine levels rise in the serum, resulting in IDO activation (7). TRP levels are decreased in the CSF and serum of patients with MS, suggesting the role of KP metabolism in MS pathogenesis (24–26). In all stages of MS, changes in the balance between neurotoxic and neuroprotective kynurenine metabolites have been observed (27). The CSF levels of HIAA are lower in MS patients compared to healthy control subjects (28). Finally, any alteration in each of the KP metabolites and enzymes can affect neurons and contribute to the MS pathogenesis and neurodegeneration. Therefore, the purpose of this systematic review is to ascertain whether altered metabolites and enzymes of KP can be measured in MS.

## Materials and methods

### Search strategy

We searched the following four databases for relevant studies published up to March 2021: PubMed, Scopus, Cochrane Database of Systematic Reviews, and Web of Science. Two authors conducted an independent search using the following query: (tryptophan OR kynurenine OR kynurenate OR kynurenic OR anthranilic OR anthranilate OR quinolinate OR quinolinic OR picolinate OR picolinic OR xanthurenic OR xanthurenate) AND (multiple sclerosis OR disseminated sclerosis). Additionally, we searched the reference lists of related articles to avoid overlooking relevant studies. All 678 papers found during the search were inserted into the Endnote software for screening. Following that, 366 duplicate publications were deleted. Subsequently, the Newcastle-Ottawa scale was used to evaluate the included studies (**Table 2**).

### Inclusion and exclusion criteria

**Figure 1:**
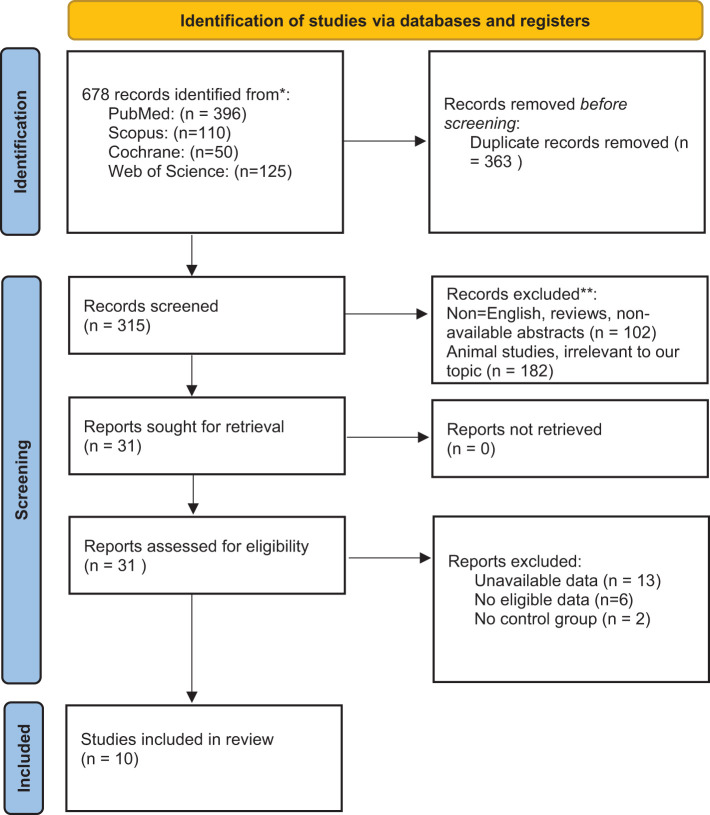
PRISMA 2020 flow diagram for new systematic reviews which included searches of databases.

We included all observational studies published in English that measured KP metabolites or enzymes in MS patients and corresponding control subjects. We excluded animal studies, those that lacked a control group, and those without randomized sampling. After excluding duplicates (366), two authors independently screened the initially identified articles based on their titles and abstracts (31 studies remained). They examined the full text of the selected studies and then shortlisted the studies that met the inclusion criteria (10). Potential disagreements were resolved by a third author (**Figure 1**).

### Data extraction

All relevant data from eligible studies were extracted, including first author name, country of origin, publication date, metabolite measurement methods, patient and control group characteristics, and measured levels of TRP, KP metabolites and enzymes. To this end, two authors extracted data independently and then compared their results. The current study was approved by the Shahid Beheshti University of Medical Sciences ethics committee IR.SBMU.RETECH.REC.1400.919.

## Results

The current study has been performed based on PRISMA checklist. After screening the titles and abstracts of the initially searched studies, 41 potentially relevant studies remained, of which 17 articles were included in the systematic review following full-text screening (3, 8, 16–18, 29–39) (**Figure 1**). It should be regarded that we also excluded some potentially relevant studies due to their designs. For instance, some studies assessed KP metabolites only in animals, and some did not compare KP metabolites levels with a healthy control group (19, 40, 41). All studies included were published in English and published up to March 2021. Two studies assessed the variables in two distinct populations (32, 39). One study utilized two cohorts, one of which met our inclusion criteria and was included in the systematic review (16). **Table 1** summarizes the study characteristics and significant findings from the included studies.

**Table 1:** Summary of the main findings from studies included in the systematic review.

Study (ref)	Place	Patients (No)			Controls (No)			Age (mean (SD))		Types of MS	Treatment	Patients phase	Materials	Methods	Metabolites	Key findings
		All	Male	Female	All	Male	Female	Patients	Controls						
Rajda C (8)	Hungary	37	18	19	22	11	11	34.10	38.60	?	None	–	CSF	Mass spectrometry	TRP, KYN, KYNA, QUIN, PIC, HIAA	No significant difference in TRP, KYNA, PIC, AND HIAA levels between the two groups, KYN and QUIN levels higher in patients
Gaetani L (31)	Italy	47	7	40	43	16	27	31.80 (9.70)	32.70 (10.60)	RRMS	11 (None), 15 (Interferons), 10 (Glatiramer acetate), 6 (Dimethylfumarate), 3 (Fingolimod), 1 (Natalizumab), 1 (Alemtuzumab)	–	urine	HPLC-Mass spectrometry/Mass spectrometry	KYN, KYN/TRP	Lower KYN levels and KYN/TRP in patients
Hartai Z (32)	Hungary	13	6	7	14	5	9	35.40 (13.10)	33.50 (11.70)	RRMS	None	1 to 3 days after the appearance of new neurological signs	plasma	Mass spectrometry	KYNA	Higher KYNA levels in patients
Hartai Z (32)	Hungary	13	6	7	14	5	9	35.40 (13.10)	33.50 (11.70)	RRMS	None		plasma	spectrophotometrically	KAT I, KAT II	No significant change in the activities of the KATs in the plasma
Hartai Z (32)	Hungary	13	6	7	14	5	9	35.40 (13.10)	33.50 (11.70)	RRMS	None		RBCs	Mass spectrometry	KYNA	No significant difference in KYNA levels between the two groups
Hartai Z (32)	Hungary	13	6	7	14	5	9	35.40 (13.10)	33.50 (11.70)	RRMS	None		RBCs	spectrophotometrically	KAT I, KAT II	Higher KAT I and KAT II activities in the RBC of the patients with MS than in the control group.
Tomosi F (39)	Hungary	20	0	20	14			33.84 (9.14)	37.57 (10.09)	RRMS	–		CSF	UHPLC– Mass spectrometry/Mass spectrometry	TRP, KYN, KYNA, QUIN, PIC, HIAA, QUIN/KYNA, KYNA/KYN, KYN/TRP	Lower levels of KYNA, PIC, and KYNA/KYN in patients. No significant difference in KYN, TRP, HIAA levels, and KYN/TRP between the two groups. Higher levels of QUIN and QUIN/KYNA in patients
Tomosi F (39)	Hungary	20	0	20	14			33.84 (9.14)	37.57 (10.09)	RRMS	–		serum	UHPLC– Mass spectrometry/Mass spectrometry	TRP, KYN, KYNA, QUIN, PIC, HIAA, QUIN/KYNA, KYNA/KYN, KYN/TRP	Lower levels of HIAA in patients. No significant difference in TRP, KYN, KYNA, PIC, KYNA/KYN, and KYN/TRP between the two groups. Higher levels of QUIN and QUIN/KYNA in patients
Mancuso R (3)	Austria	36	13	23	15	5	10	37.94 (8.52)	37.83 (9.55)	RRMS	Glucocorticoid treatment for AMS patients	21 AMS 15 SMS	Serum	HPLC	TRP, KYN, KYN/TRP	No significant difference in TRP, KYN, and KYN/TRP between SMS patients and HCs. KYN levels and KYN/TRP were significantly higher in SMS patients, AMS patients before the initiation of glucocorticoids, and HCs compared with AMS patients after the initiation of glucocorticoids.
Mancuso R (3)	Austria	36	13	23	15	5	10	37.94 (8.52)	37.83 (9.55)	RRMS	Glucocorticoid treatment for AMS patients	21 AMS 15 SMS	PBMCs	spectrophotometrically	IDO mRNA	IDO expression was decreased in SMS patients compared to HCs and AMS patients before the initiation of glucocorticoids. IDO expression was higher in SMS patients than AMS patients after the initiation of glucocorticoids.
Nergotto L (35)	Argentina	40	13	27	30	10	20	32.00 (7.90)	32.00 (5.90)	RRMS	16 (IFN-β1a) and 9 (glatiramer acetate)	Remission	PBMCs	reversed phase HPLC	TRP, KYN	Higher levels of TRP in patients, Lower levels of KYN in patients
Nergotto L (35)	Argentina	40	13	27	30	10	20	32.00 (7.90)	32.00 (5.90)	RRMS	16 (IFN-β1a) and 9 (glatiramer acetate)	Remission	PBMCs	Real time PCR, ELISA	IDO mRNA, IDO protein	Reduced levels of IDO expression in patients both at mRNA and protein levels
Aeinehband S (16)	Sweden	86	34	52	20	8	12	43.30 (11.80)	36.50 (9.30)	72 RRMS, 5 PPMS, and 9 SPMS	77 (None), 7 (interferons), 1 (rituximab), 1 (glatiramer acetate)	8 AMS, 19 SMS	CSF	HPLC- Mass spectrometry/Mass spectrometry	TRP, KYN, KYNA, QUIN, QUIN/KYNA, KYNA/KYN, KYN/TRP	Significant lower levels of TRP, KYN, KYNA, QUIN, KYN/TRP, and KYNA/KYN in MS patients compared with iOND patients. No significant difference in QUIN/KYNA between MS and iOND patients. Significant lower levels of QUIN/KYNA in RRMS-relapse patients compared with OND patients. No significant difference in TRP, KYN, KYNA, QUIN, KYN/TRP, and KYNA/KYN between RRMS-relapse patients and OND patients. The levels of TRP and KYNA were significantly higher in PPMS patients than in SPMS and RRMS patients. The levels of QUIN and KYNA were significantly higher in PPMS patients than in OND patients.
Agliardi C (30)	Italy	675	235	440	680	271	409	50.0	64.17	596 RRMS, 79 PPMS	–	–	Blood	Spectrophotometrically	IDO2 mRNA expression	No significant differences in IDO2 activity between multiple sclerosis patients and HCs
Huang YM (34)	Sweden	37	0	37	37	0	37	31.59 (6.54)	31.19 (6.27)	RRMS	None	Remission	PBMCs	Spectrophotometrically	IDO mRNA expression	Lower levels of IDO mRNA expression in all the Multiple sclerosis patients compared to all HCs
Nejati A (36)	Iran	84	24	60	70	20	50	34.55 (8.83)	34.16 (8.26)	RRMS	7 (None), 74 (Interferon), 1 (Zidovudine), 1 (Mitoxantrone), 1 (Glatiramer acetate)	–	PBMCs	Spectrophotometrically	IDO mRNA expression	Lower levels of IDO mRNA expression in Multiple sclerosis patients compared to HCs
Lim (42)	Australia	87	29	58	49	14	35	47.44 (10.39)	45.29 (11.7)	50 RRMS, 20 SPMS, 17 PPMS	–		Serum, CSF	UHPLC	TRP, KYN, KYNA, KYN/TRP	Increased KYN/TRP in the serum of all the MS subtype groups, higher KYNA serum levels in the RRMS group relative to HCs and progressive MS groups
Olsson (37)	Denmark	58	14	44	50	16	34	34	33	RRMS	None	Before initiation of the first disease modifying therapy	Serum	Mass spectrometry	TRP, KYN, KYNA, IDO	Lower KYNA levels in MS patients, No differences in IDO1 expressions between the two groups
Sadowska-bartosz (38)	poland	60			18			26-50	26-45	RRMS, SPMS	INF β1a, INF β1b, mitoxantrone		Serum	Fluorescence assessment	TRP, KYN	KYN increased in RRMS patients without treatment and RRMS patients treated with IFN-β1b
Rejdak (17)	poland	26								RRMS, SPMS	None	Remission	CSF	HPLC	KYNA	Lower KYNA levels in MS patients compared with patients with non-inflammatory neurological disorders
Rejdak (18)	poland	20	6	14	10	4	6	28	29	RRMS	None	Relapse	CSF	HPLC	KYNA	The CSF KYNA was higher in the RRMS group
Herman (33)	sweden	46			10			45.6 (13.6)	39	16 SPMS, 30 RRMS	–		CSF	HPLC	KYN	SPMS patients had higher KYN levels compared with healthy controls, and RRMS patients
Adamczyk-sowa (29)	poland	14	7	7	11	5	6	40.65(10.01)	34.54 (9.6)	RRMS	IFNβ, melatonin		Serum	Fluorescence assessment	KYN	Levels of KYN were elevated in non-treated RRMS

CSF, cerebrospinal fluid; RBC, red blood cell; PBMCs, peripheral blood mononuclear cell; HPLC, High-performance liquid chromatography; UHPLC, Ultra-high performance liquid chromatography; TRP, tryptophan; KYN, kynurenine; KYNA, kynurenic acid; QUIN, quinolinic acid; PIC, picolinic acid; HIAA, hydroxyindoleacetic acid HCs, healthy controls; PCR, polymerase chain reaction; ELISA, enzyme-linked immunosorbent assay; AMS, MS patients in acute phase; SMS, MS patients in stable phase.

### Kynurenine

Eleven studies (3, 8, 16, 29, 31, 33, 35, 37–39, 42) involving 730 individuals provided data on KYN levels (468 MS patients and 262 healthy controls). Three studies (8, 16, 33) used CSF as the sample source, four studies utilized serum (3, 29, 37, 38), one study employed peripheral blood mononuclear cells (PBMCs) (35), one study used urine as the sample source (31), and two studies utilized both CSF and serum (39, 42). Negrotto et al. (35) reported that RRMS patients in the remission phase had remarkably lower KYN levels than controls in PBMCs (P< 0.001). Moreover, according to a study by Gaetani et al. (31), RRMS patients had significantly lower KYN levels than controls in the urine sample (P= 0.010). In contrast, a study conducted by Rajda et al. (8) illustrated that MS patients had significantly higher levels of kynurenine in their CSF than healthy controls (P=0.049). Moreover, Sadowska-bartosz et al. (38) and Adamczyk-sowa et al. (29) stated in their studies that RRMS patients without treatment have considerably increased levels of KYN than healthy controls in their serum (P<0.05). Additionally, Sadowska-bartosz et al. (38) and Adamczyk-sowa et al. (29) reported significantly elevated levels of KYN in the serum of RRMS patients without treatment compared with RRMS patients treated with IFN-β1b (P<0.05 and P<0.01, respectively). Besides, Herman et al. (33) illustrated that SPMS patients had remarkably higher KYN levels in their CSF in comparison to healthy controls and RRMS patients (P<0.05). Also, in studies by Aeinehband et al. (16) on CSF (p>0.05), Mancuso et al. (3) on serum (P>0.05), Lim et al. (42) on serum and CSF, Olsson et al. on serum, and Tomosi et al. (39) on both CSF and serum samples (P=0.169 and P= 0.894, respectively), no significant difference in kynurenine levels was observed between MS patients and controls. ()

### Tryptophan

Eight studies (3, 8, 16, 35, 37–39, 42) involving 559 individuals (361 MS patients and 198 healthy controls) provided data on TRP levels. Two studies (8, 16) used CSF as the sample source, three studies utilized serum (3, 37, 38), one study used PBMCs as the sample source (35), and two studies employed both CSF and serum (39, 42). Negrotto et al. (35) reported significantly higher TRP levels in the PBMCs of RRMS patients who are in the remission phase compared to controls (p=0.0007). However, in a study by Rajda et al. (8), CSF levels of TRP were insignificantly lower in MS patients than in healthy controls (p=0.12). Three other studies by Aeinehband et al. (16), Tomosi et al. (39), and Lim et al. (42) demonstrated no significant differences in the CSF levels of TRP in MS patients in comparison to controls (p>0.05, p=0.92, and P>0.05 respectively). Similarly, five studies conducted by Tomosi et al. (39), Mancuso et al. (3), Lim et al. (42), Olsson et al. (37), and Sadowska-bartosz et al. (38) reported that TRP levels in the serum samples of MS patients did not change significantly (p> 0.05).

### Kynurenic acid

Eight studies (8, 16–18, 32, 37, 39, 42) collected comparative data on KYNA levels in 478 individuals (299 patients and 179 controls). Four studies (8, 16–18) used CSF as the source of the sample; two studies utilized both CSF and serum (39, 42), one study used serum samples (37), and one study employed both red blood cells (RBCs) and plasma as the source of the sample (32). Rejdak and his colleagues (17) reported in a study that KYNA levels in the CSF of MS patients in the remission phase are lower than in patients with non-inflammatory neurological disorders (P<0.01). However, in another study by Rejdak et al. (18), they reported significantly elevated KYNA levels in the CSF of RRMS patients who are in the relapse phase (P=0.01). Also, KYNA levels in the CSF were significantly lower in RRMS patients than in controls in the studies conducted by Tomosi et al. (39) (p=0.04). In a study done by Olsson et al. (37) it was shown that RRMS patients without any treatment had lower serum KYNA concentrations (P<0.05), but there were no significant differences between MS patients and controls in the studies performed by Aeinehband et al. (16) and Rajda et al. (8) (p>0.05 and p=0.42, respectively). On the other hand, Hartai et al. (32) discovered that MS patients had a remarkably higher level of KYNA in their plasma than controls (p<0.05). Additionally, Lim et al. revealed that RRMS patients have higher serum KYNA in comparison to healthy controls and progressive MS patients (P<0.0001). However, studies were done by Tomosi et al. (39) on serum, and Hartai et al. (32) on RBCs, reported no significant difference in KYNA levels between MS patients and healthy controls (p=0.16 and p>0.05, respectively).

### Quinolinic acid

Three studies (8, 16, 39) collected data on QUIN levels in 151 individuals (95 MS patients and 56 healthy controls). In two studies (8, 16), the sample source was CSF; in one study, the sample source was both CSF and serum (39). Rajda et al. (8) and Tomosi et al. (39) showed that the QUIN levels in CSF were remarkably higher in MS patients than in healthy controls (p=0.001 and p<0.0001, respectively). Likewise, Tomosi et al. (39) reported a higher level of QUIN in serum samples of MS patients compared with controls (p=0.030). Conversely, there was no significant difference in the CSF levels of QUIN in a study conducted by Aeinehband et al. (16) (p>0.05).

### Picolinic acid

Two studies (8, 39) collected data on the picolinic acid (PIC) levels of 93 individuals (75 MS patients and 18 controls). In one study (8), the sample was obtained from CSF, while in another study, the sample was obtained from both CSF and serum (39). Tomosi et al. found that PIC levels were significantly lower in the CSF sample of MS patients than in healthy controls (39) (p=0.02). However, according to Rajda et al. (8) and Tomosi et al. (39), there was no significant difference in PIC levels of CSF and serum samples, respectively, between MS patients and controls (p=0.59 and p=0.25, respectively).

### Kynurenine, tryptophan, quinolinic acid ratio

Five studies measured KYN/TRP ratios (3, 16, 31, 39, 42) in 354 individuals (213 MS patients and 141 healthy controls). One study used urine as the sample source (31), one used CSF (16), two used both serum and CSF (39, 42), and the last study used serum samples (3). It has been elucidated by Lim et al. (42) that KYN/TRP was significantly increased in the serum samples of all the MS subtype groups (RRMS, PPMS, and SPMS) compared to healthy controls (P<0.0001). Also, Mancuso et al. (3) showed that KYN/TRP was higher in MS patients in the stable phase, MS patients in the acute phase before the initiation of glucocorticoids, and healthy controls compared with AMS patients after the initiation of glucocorticoids (P<0.05). Gaetani et al. (31) found that MS patients had remarkably lower KYN/TRP ratio in urine samples than the controls (p=0.04). In three studies were done by Aeinehband et al. (16), Mancuso et al. (3), and Tomosi et al. (39), no significant differences in KYN/TRP ratios were observed between MS patients and controls in CSF or serum samples (3, 16, 39).

Two studies (16, 39) assessed QUIN/KYNA ratios in 92 individuals (58 MS patients and 34 healthy controls). In one study, the sample source was CSF (16), while the other study used serum and CSF (39). According to the result of the study by Tomosi et al. (39), QUIN/KYNA ratios were significantly higher in MS patients compared to healthy controls in both CSF and serum samples (p=0.0015 and p=0.0183, respectively). However, Aeinehband et al. (16) reported that there was no significant difference in CSF QUIN/KYNA ratios between MS patients and controls (p>0.05).

Two studies (16, 39) involving 92 participants determined the KYNA/KYN ratio (58 MS patients and 34 healthy controls) in MS patients and controls. Tomosi et al. (39) discovered that MS patients had significantly lower KYNA/KYN ratios compared to controls when CSF samples were analyzed (p=0.0041), but there was no significant difference when serum samples were measured (p=0.0832). Aeinehband et al. (16) reported no statistically significant difference in KYNA/KYN ratios between MS patients and controls (p>0.05).

### Indoleamine 2,3-dioxygenase

The expression of IDO mRNA was analyzed in six studies (3, 30, 34–37), including 1812 individuals (930 MS patients and 882 healthy controls). five studies (3, 30, 34–36) assessed IDO mRNA in mononuclear cells. One study (37) used whole blood for assessment. Four studies were done by Huang et al. (34), Mancuso et al. (3), Negrotto et al. (35), and Nejati et al. (36) reported reduced levels of IDO mRNA expression in MS patients in comparison to controls (p<0.05, p=0.01, p<0.001, and p<0.0001 respectively). However, Agliardi et al. (30) and Olsson et al. (37) showed no difference in IDO mRNA expression between MS patients and healthy controls. Additionally, Negrotto et al. (35) measured IDO1 protein levels in PBMCs and detected reduced IDO1 protein expression in MS patients when compared to healthy controls (p<0.001).

### Kynurenine aminotransferase

One study, including 27 individuals (13 MS patients and 14 healthy controls) measured the enzymatic activity of both KAT I and KAT II in plasma and RBCs (32). It showed that the activities of both KAT I and KAT II enzymes are significantly higher in RBCs of MS patients compared with healthy controls (p<0.05). However, no significant difference in KAT I and KAT II plasma enzymatic activity could be detected between MS patients and healthy controls.

## Discussion

In recent years, there has been mounting evidence that KP plays a significant role in neurodegenerative diseases such as MS (27). Inflammation or degeneration of the CNS induces the metabolism of TRP primarily through the production of KYN and related breakdown products (43). As MS progresses, levels of inflammatory cytokines, including interferon-γ (IFN-γ) and Tumor necrosis factor-α (TNF-α), increase, activating KP (44, 45). This study systematically reviewed 10 published primary research articles investigating differences between MS patients and healthy controls in serum, CSF, and urine levels of six major metabolites and two enzymes associated with the KP. We focused on TRP, KYN, KYNA, QUIN, and PIC levels as well as IDO mRNA expression and KAT activity.

MS pathogenesis likely involves several different mechanisms. One of the most popular hypotheses is that the infiltration of immune-activated macrophages and T cells causes death of oligodendrocytes that are responsible for myelinating axons in a healthy CNS (46, 47). KP metabolites have been suggested to promote both immune tolerance and autoimmunity according to this model of MS pathogenesis. Studies have revealed significantly lower TRP levels in the serum and CSF of MS patients, suggesting that KP activation may play a role in the disease pathogenesis (24, 25). In the human CNS, TRP is mostly metabolized through KP. Nevertheless, there are cells that do not express the entire enzymatic pathway. Only reactive microglia, infiltrating macrophages, and active neurons contain the complete pathway (43, 48). A study using urine samples from 47 Relapsing Remitting Multiple Sclerosis (RRMS) patients and 43 healthy controls reported that women had lower levels of urinary TRP and KYN than men (31). After adjusting for age and gender, urine concentrations of TRP did not show a significant difference between the RRMS and control group. Although the expanded disability scale (EDSS) has shown significant correlation with TRP urinary concentrations, disease duration has not been associated with KP metabolite levels (31). In contrast, another study reported significantly higher levels of TRP in PBMCs of RRMS patients compared to healthy controls (35). Aeinehband et al. (16) investigated cross-sectional cell-free CSF samples from patients with RRMS in both the relapse and remission phases, Primary-Progressive Multiple Sclerosis (PPMS), Secondary-Progressive Multiple Sclerosis (SPMS), for KP metabolites, using patients living with other neurological diseases, including syringomyelia, vertigo, anxiety, postcommotio syndrome, alcohol-related spastic paraparesis, neurasthenia, and unspecific sensory symptoms, as controls. They found that although there was no absolute difference in CSF concentrations of KP metabolites between PPMS and SPMS patients, PPMS patients displayed increased levels of all metabolites except for TRP in comparison to SPMS patients (16). In addition, disparities in TRP concentrations could be associated with variable characteristics of the enrolled patients reflecting the correlation between disease activity as well as disease courses with changes in KP metabolites. Moreover, inflammatory processes that initiate KP metabolism are associated with fluctuations in cytokine concentrations throughout the various phases of MS (3), which may contribute to the controversial results reported in recent publications. Future studies should compare concentrations of KP metabolites and MS disease activity in order to find novel therapeutic targets and prognostic markers.

The conversion of TRP to KYN is the first and rate-limiting step in KP metabolism, and is regulated by IDO-1 in most human tissues and TDO in liver cells (49). There have been multiple studies indicating that KYN influences the proliferation of several T cell subtypes, including CD4^+^ T lymphocytes and CD8^+^ T lymphocytes (50–52). In addition, it has been demonstrated that KYN can compromise the function of natural killer cells while simultaneously showing pro-apoptotic effects (53, 54). Therefore, KYN levels have been measured in the serum, CSF, PBMCs, and urine of MS patients and compared to healthy controls. There have been substantial differences among published results. RRMS patients had considerably lower urinary KYN concentrations when compared to healthy controls (31); however, KYN concentrations in CSF did not differ significantly between MS and non-inflammatory neurological disorders patients (16). When RRMS patients treated with IFN-β were compared to untreated RRMS patients, an increase in KYN level was observed (29, 55, 56). In contrast, another study did not find any alterations of KP activation resulting from IFN-β therapy in untreated MS patients (16).

The KYN/TRP ratio is indicative of the IDO activity as well as KP. IDO expression could potently be induced by several mediators including IFN-γ, TGF-β, toll-like receptor ligands, polyamines, TNF-α, platelet activating factor, and human immunodeficiency virus (HIV) proteins (51, 57–61). IDO mRNA expression was found to be lower in MS patients compared to healthy controls (3, 31, 34–36). Lower KYN along with a decreased urine KYN/TRP ratio in RRMS patients was found to be inversely related to the intensity of disability, suggesting a reduced TRP metabolism in the earliest stages of the disease (31, 62). Contrarily, some studies determined a significantly increased KYN/TRP ratio in MS patients compared to healthy controls (42), in addition to other studies demonstrating no meaningful difference in KYN/TRP ratios between MS patients and healthy subjects (3, 16, 39). These discrepancies could result from different biofluid samples analyzed, indicating different phases of TRP metabolism and variation in KP enzymes involved in each site. Moreover, the treatment that MS patients receive may affect the KP metabolites. For example, in the study conducted by Gaetani et al. (31), most MS patients received MS treatment, especially interferons. Thus, to elucidate the variation in KP metabolite concentrations in different organs and tissues, further controlled studies on different body fluids such as urine, blood, and CSF concurrently in the same subjects are needed.

Enzyme KAT converts KYN to KYNA (63). The KYNA/KYN ratio was increased in the CSF of MS patients compared to controls, while no difference was detected in serum ratios from the same subjects (39). Authors, in line with previous studies, also demonstrated lower CSF levels of KYNA among MS patients compared to healthy controls (17). Lim et al. demonstrated decreases in the levels of both enzymes that produce KYNA in the postmortem MS brain sections, correlating with lower levels of KYNA (64). In disagreement, KYNA concentrations were found to be significantly higher in the plasma and cerebrospinal fluid of patients with MS compared to healthy subjects. Additionally, researchers have stated that KYNA has a neuroprotective role in progressive MS (32, 65). PPMS patients are unique in having significantly increased levels of KYNA, which has been found to display neuroprotective effects both experimentally and clinically, decelerating disease progression (66, 67). Further research has revealed elevated KYNA levels during the acute relapse phase of MS (18). Conversely, SPMS patients show a decreased neuroprotection index (68), confirming the idea of altered KP activation among patients with different MS clinical courses.

**Figure 2:**
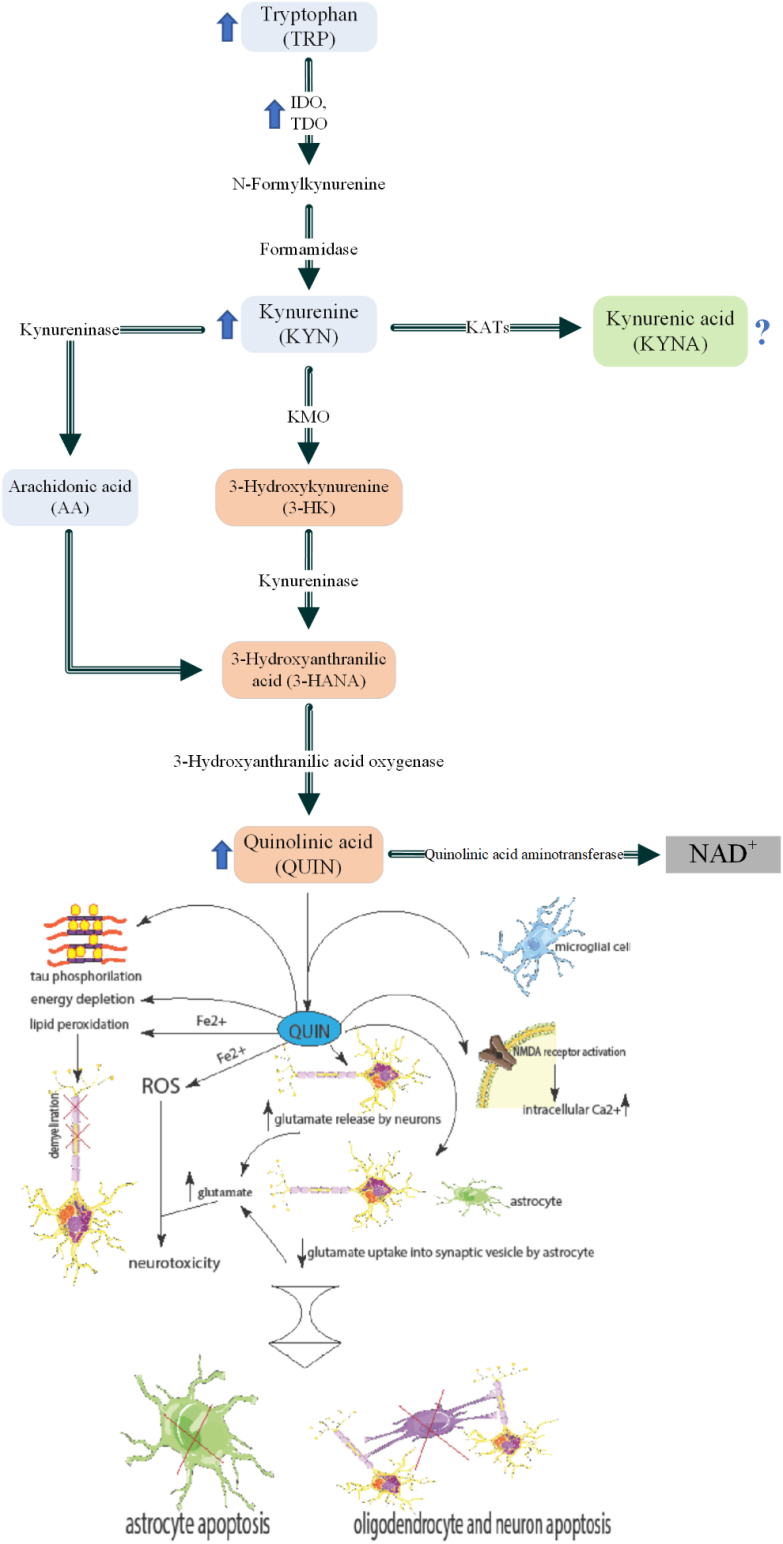
Kynurenine pathway and roles of QUIN in MS pathogenesis. Inflammatory cytokines such as TNF-α and INF-α which are increased in MS patients blood circulation. These induce increased activity and levels of IDO and KMO in macrophages. Higher activity of these enzymes leads to KP activation and thus high levels of QUIN is secreted to the blood. The increased QUIN can pass the BBB and enter the brain parenchyma. This QUIN is together with the QUIN secreted by microglia lead to several pathological mechanisms: 1) NMDA receptor activation in the cells and therefore higher intracellular calcium. 2) increased glutamate release by neurons and decreased glutamate uptake into synaptic vesicles by astrocytes. These cause higher glutamate levels in the micro-environment which cause neurotoxicity. 3) Increased ROS formation which causes neurotoxicity. 4) Lipid peroxidation that can lead to demyelination. 5) Energy depletion 6) Tau phosphorylation. The mentioned mechanisms, generally cause apoptosis of astrocytes, oligodendrocytes (an important cell in myelin production), and neurons. Parts of the figure were drawn by using pictures from Servier Medical Art. Servier Medical Art by Servier is licensed under a Creative Commons Attribution 3.0 Unported License (https://creativecommons.org/licenses/by/3.0/).

In contrast to neuroprotective KYNA, QUIN is considered a neurotoxic metabolite of KP (6, 6, 19), thus, shifting KP toward KYNA instead of QUIN could be a potential therapeutic strategy. Although astrocytes do not utilize the full enzymatic pathway, they produce high levels of KYN that can be metabolized to QUIN by microglia, monocytes, or infiltrating macrophages and result in neurotoxicity (69–71). The higher QUIN/KYN ratio during the relapse phase of MS patients compared with remission phase, confirms that the QUIN-induced apoptosis of myelin producing oligodendrocytes as a sign of failed remyelination (16, 68, 69, 72). Furthermore, QUIN was found to be responsible for the impaired phosphorylation of tau protein in progressive MS (73). Indeed, QUIN could be a potential biomarker of active relapse and demyelinating phases of MS (**Figure 2**).

**Table 2:** Evaluation of quality of included studies using the QUADOMICS tool.

Study (ref)	1	2	3	4.1	4.2	5	6	7	8	9	10	11	12	13	14	15	16
Rajda C (8)	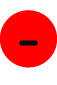	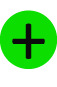	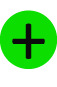	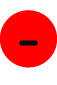	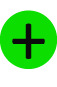	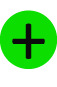	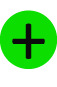	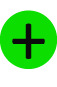	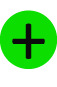	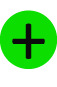	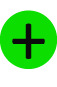	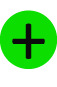	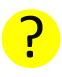	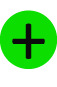	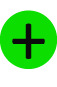	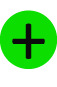	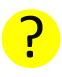
Gaetani L (31)	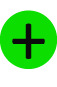	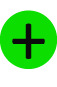	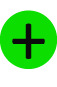	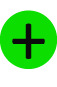	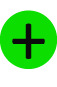	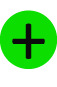	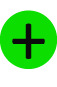	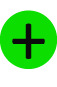	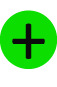	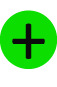	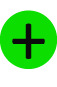	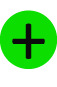	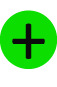	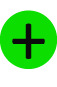	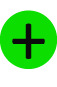	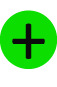	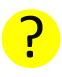
Hartai Z (32)	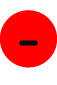	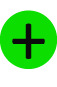	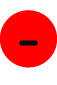	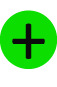	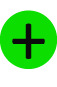	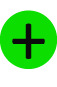	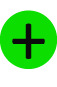	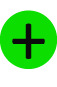	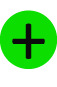	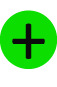	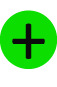	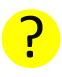	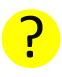	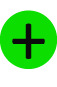	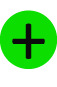	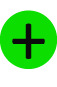	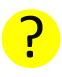
Tomosi F (39)	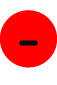	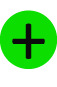	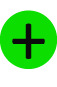	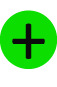	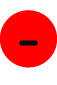	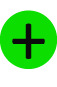	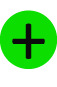	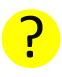	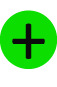	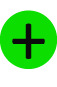	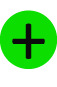	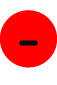	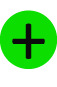	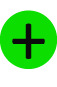	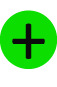	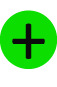	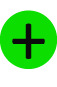
Mancuso R (3)	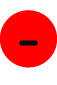	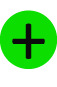	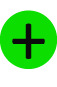	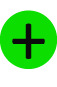	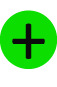	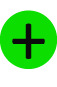	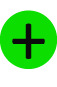	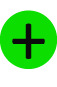	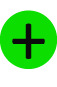	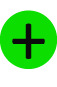	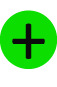	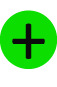	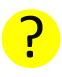	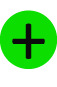	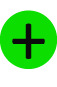	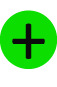	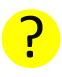
Nergotto L (35)	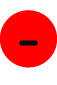	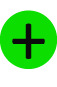	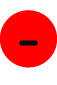	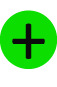	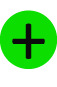	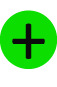	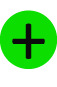	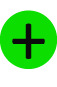	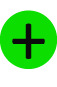	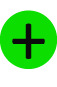	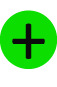	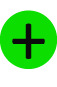	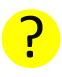	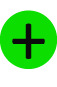	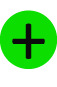	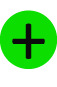	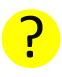
Aeinehband S (16)	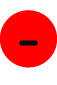	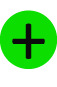	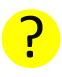	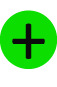	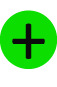	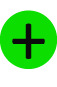	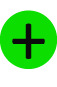	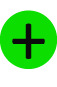	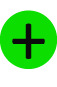	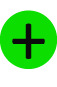	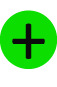	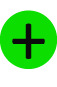	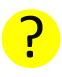	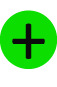	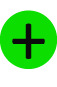	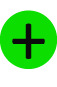	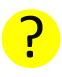
Agliardi C (30)	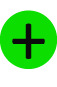	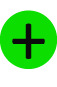	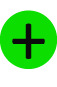	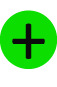	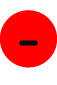	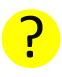	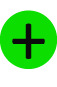	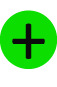	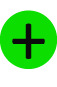	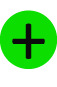	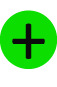	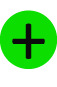	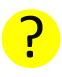	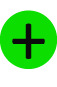	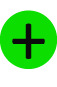	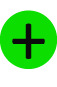	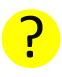
Huang YM (34)	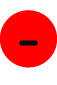	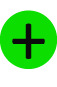	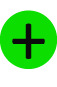	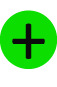	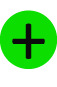	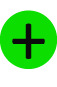	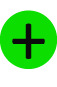	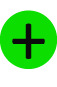	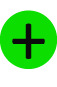	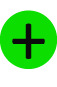	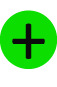	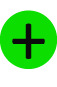	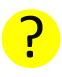	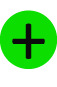	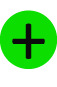	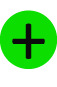	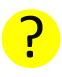
Nejati A (36)	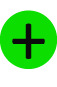	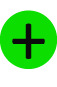	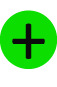	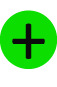	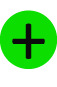	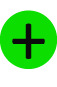	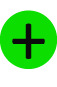	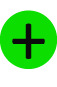	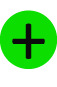	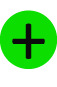	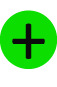	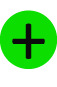	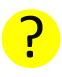	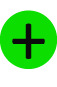	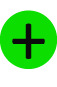	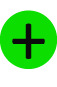	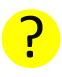
Lim (42)	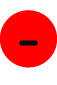	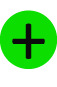	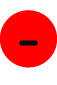	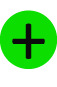	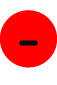	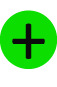	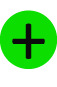	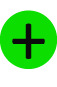	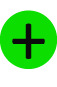	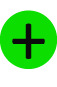	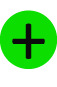	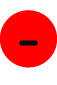	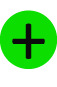	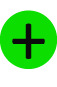	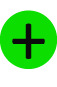	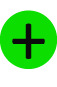	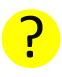
Olsson (37)	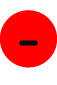	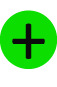	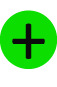	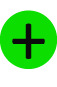	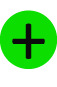	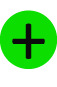	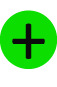	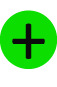	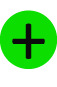	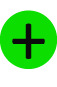	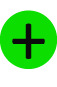	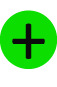	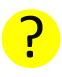	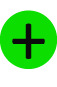	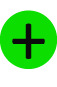	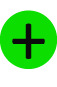	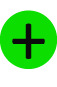
Sadowska-bartosz (38)	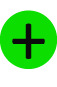	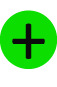	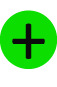	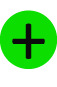	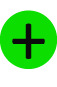	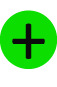	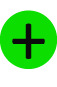	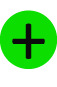	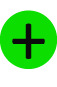	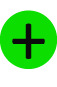	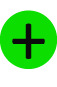	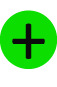	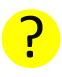	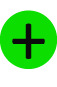	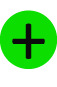	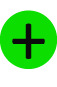	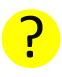
Rejdak (17)	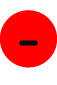	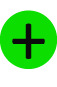	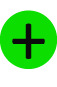	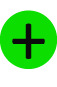	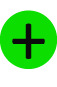	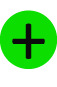	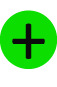	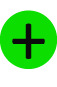	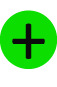	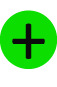	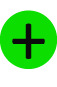	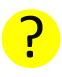	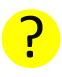	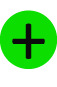	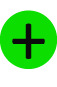	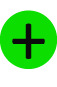	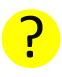
Rejdak (18)	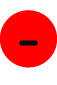	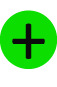	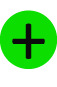	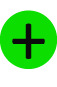	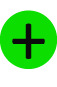	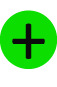	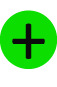	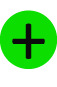	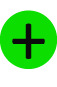	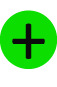	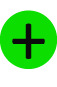	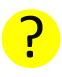	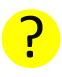	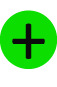	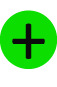	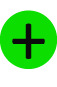	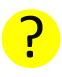
Herman (33)	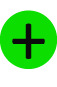	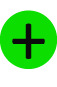	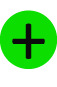	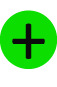	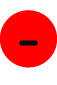	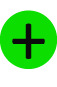	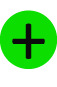	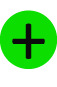	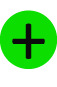	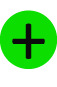	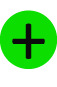	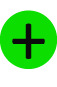	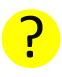	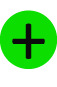	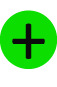	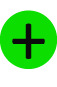	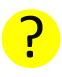
Adamczyk-sowa (29)	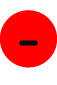	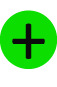	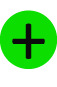	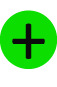	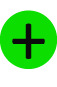	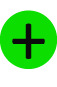	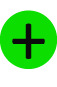	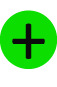	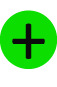	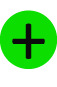	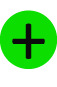	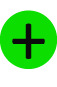	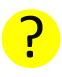	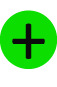	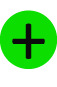	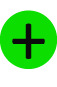	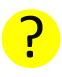

Very few studies have measured PIC in MS patients and controls. PIC induces inflammatory macrophage proteins in association with IFN-γ at low concentrations and acts as an activator of macrophages (74–76). This process of macrophages co-activation by PIC emphasizes the importance of PIC neuroprotection in neurodegenerative conditions (77). Decreased PIC levels in MS are consistent with its protective role in this and other degenerative disorders. Notably, another study demonstrated the inverse relationship between PIC and QUIN, with PIC being higher in RRMS groups but lower in PPMS groups (78).

In summary, the importance of KP metabolites as prognostic, diagnostic, and therapeutic biomarkers is commonly known. It is still unknown whether KP is beneficial in the pathogenesis of MS by acting as a protective pathway or whether its activation is a sign of deterioration; however, it is well established that prolonged KP metabolism and the accumulation of neurotoxic metabolites accelerate the progression of MS. More controlled studies on specific fluid samples from particular disease phases are needed to unravel the changes the KP undergoes during MS pathogenesis. Further research is necessary to evaluate the KP metabolism rate and its possible subtypes in patients with MS at all stages while also considering demographic data of patients, including age and sex. Moreover, due to changes in KP during different phases of MS and in different types of MS and the effect of MS treatment on KP, it is suggested to report data about the type and phase of MS in patients and the treatment that they have been received when measuring the KP metabolites. Moreover, given the effect of disease-modifying therapies such as IFN-β1 on KP metabolite levels and the effect of KP activation on treatment efficacy, additional research should focus on the effect of available therapies on KP metabolite concentrations and their effects on treatment efficacy.

## Limitations

Our study has important limitations. First, reported details of patient characteristics were limited, consequently, findings could not be conclusively extrapolated to MS in general. Second, only a small number of selected articles met our criteria for covering all MS stages. This could be one of the reasons for the discrepancies mentioned above. Third, the studies that were investigated included samples collected from different tissues, which made it difficult to comprehensively compare the results.

## Conclusion

In conclusion, this study established a potential link between altered KP metabolite levels and MS disease progression. Based on our systematic review, different KYN metabolites can be measured in MS, highlighting the potential role of KP in the pathophysiology of MS. This finding is critical for future research, which would benefit from larger scale studies comparing KP metabolites in individuals with MS. QUIN has previously been suggested to contribute to neurodegeneration. In this review we found that QUIN levels in the CSF of MS patients was higher compared to healthy controls, indicating that QUIN may play a role in MS pathogenesis. Although it was suggested that KYNA is neuroprotective and have beneficial effects in MS, the difference of KYNA levels between MS patients and controls was not significant. Also, different levels of other KP metabolites, including KYN, TRP, PIC and their ratio were also found between MS patients and controls; however, there were discrepancies between studies. Further high-quality studies on peripheral and central KP metabolite concentrations are required to better understand the dynamics of these metabolite levels in MS. Further research is also necessary to overcome our study limitations and to evaluate the rate of KP metabolism and its possible subtypes in patients with MS at all stages and ages. Moreover, given the effect of disease-modifying therapies such as IFN-β1 on KP metabolite levels and the effect of KP activation on treatment efficacy, additional research should focus on the effect of available therapies on KP metabolite concentrations and their likely effects on treatment efficacy.

## Data availability statement

The original contributions presented in the study are included in the article/supplementary material. Further inquiries can be directed to the corresponding authors.

## Author contributions

MF, KV, and SY contributed to the conception and design of the study. MH and FS contributed to the supervision of the manuscript. SY organized the database. AK and AMc edited the paper scientifically. All authors contributed to the article and approved the submitted version.

## Acknowledgments

The authors of this study are thankful to the Clinical Research Development Center (CRDC) and Skull Base Research Center of Loghman Hakim Hospital, Shahid Beheshti University of Medical Sciences, Tehran, Iran for their support, cooperation, and assistance throughout the period of study.

## Conflict of interest

The authors declare that the research was conducted in the absence of any commercial or financial relationships that could be construed as a potential conflict of interest.

## Publisher’s note

All claims expressed in this article are solely those of the authors and do not necessarily represent those of their affiliated organizations, or those of the publisher, the editors and the reviewers. Any product that may be evaluated in this article, or claim that may be made by its manufacturer, is not guaranteed or endorsed by the publisher.

## Abbreviations

CSF, cerebrospinal fluid; MS, multiple sclerosis; RBC, red blood cell; PBMC, peripheral blood mononuclear cell; TRP, tryptophan*;* KYN, kynurenine*;* KYNA, kynurenic acid*;* QUIN, quinolinic acid*;* PIC, picolinic acid*;* HIAA, hydroxyindoleacetic acid*;* LC, Liquid chromatography; GC, gas chromatography; IDO,Indolaeamine 2,3-Dioxygenase; HK,3-hydroxykynurenine; AA, anthranilic acid; XA, xanthurenic acid; KMO, Kynurenine 3 Monooxygenase; ROS, Reactive oxygen species; BBB, Blood brain barrier.
